# The genus *Borrelia* reloaded

**DOI:** 10.1371/journal.pone.0208432

**Published:** 2018-12-26

**Authors:** Gabriele Margos, Alex Gofton, Daniel Wibberg, Alexandra Dangel, Durdica Marosevic, Siew-May Loh, Charlotte Oskam, Volker Fingerle

**Affiliations:** 1 Bavarian Health and Food Safety Authority and National Reference Center for Borrelia, Oberschleissheim, Germany; 2 Vector & Waterborne Pathogens Research Group, School of Veterinary & Life Sciences, Murdoch University, South St, Murdoch, Australia; 3 Cebitec, University of Bielefeld, Bielefeld, Germany; Umeå University, SWEDEN

## Abstract

The genus *Borrelia*, originally described by Swellengrebel in 1907, contains tick- or louse-transmitted spirochetes belonging to the relapsing fever (RF) group of spirochetes, the Lyme borreliosis (LB) group of spirochetes and spirochetes that form intermittent clades. In 2014 it was proposed that the genus *Borrelia* should be separated into two genera; *Borrelia* Swellengrebel 1907 emend. Adeolu and Gupta 2014 containing RF spirochetes and *Borreliella* Adeolu and Gupta 2014 containing LB group of spirochetes. In this study we conducted an analysis based on a method that is suitable for bacterial genus demarcation, the percentage of conserved proteins (POCP). We included RF group species, LB group species and two species belonging to intermittent clades, *Borrelia turcica* Güner et al. 2004 and *Candidatus* Borrelia tachyglossi Loh et al. 2017. These analyses convincingly showed that all groups of spirochetes belong into one genus and we propose to emend, and re-unite all groups in, the genus *Borrelia*.

## Introduction

The spirochete genus *Borrelia*, named after the French biologist Amédée Borrel, was originally described in 1907 by Swellengrebel [1], with *B*. *anserina* (Sakharoff 1891) Bergey *et al*. 1925 designated as the type species. Since then numerous species and strains have been described, and members of this genus are well recognized as the aetiological agents of Lyme borreliosis (LB) and relapsing fever (RF) in humans. Lyme borreliosis and RF genospecies have long been recognized to have different clinical, biological, and epidemiological characteristics, and phylogenetic data is concordant with this, demonstrating that these two groups are genetically similar yet distinct, and form independent monophyletic sister clades that share a common ancestor [2].

Nevertheless, LB and RF *Borrelia* share a common set of genetic and biological characteristics that unify these organisms as a group compared to other related spirochetes. Namely, all LB and RF *Borrelia* species are spirochetes with an obligate parasitic lifestyle, are transmitted between vertebrate hosts by arthropod vectors (ticks and louse), and can be transstadially transmitted within their arthropod vectors. Various vector associations of *Borrelia* have been found in nature, with the genus *Ixodes* mainly vectoring LB species while argasid ticks often vector the RF group. However, some members of the RF group are associated with hard ticks of the family Ixodidae (e.g. *B*. *lonestari*) [3], with the human body louse *Pediculus humanus* (*B*. *recurrentis*) [4] or the genus *Ixodes* (e.g. *B*. *miyamotoi*) [5]. The genus *Ixodes* represents an ancient genus of the family Ixodidae sharing numerous original features with argasid ticks. Both, LB and RF spirochetes are dependent on their vertebrate and arthropod hosts for the majority of their nutritional requirements, and share a unique genomic structure comprised of a single highly conserved linear chromosome and numerous extrachromosomal linear and circular plasmids that can be highly variable between strains [6–8].

Recently, a third group of *Borrelia* organisms has been described that are associated with reptile and echidna (*Tachyglossus aculeatus*) hosts, and do not phylogenetically cluster within either the RF or LB clades. Instead these novel borreliae form their own independent lineages which sit as an outgroup to, and shares a most recent common ancestor with, the RF clade [9]. This novel clade currently has two designated species, *B*. *turcica* [10] and ‘*Candidatus* Borrelia tachyglossi’ [9, 11, 12], and several other genetic variants that are yet to be formally taxonomically classified [13]. Known vectors for this group include hard ticks of the genera *Amblyomma*, *Bothriocroton*, and *Hyalomma* [10, 11, 13].

Recently Adeolu and Gupta [14] proposed to divide *Borrelia* into two genera to reflect the genetic and phenotypic divergence between LB and RF species, however, this proposal remains under debate [15, 16], and has not been widely utilized in the literature. The justification for this proposal was largely based on the identification of conserved signature insertions/deletions (indels) (CSIs) and conserved signature proteins (CSPs) that are differentially present in the LB or RF *Borrelia* genogroup, as well as average nucleotide identity (ANI) values calculated between whole genomes of 18 *Borrelia* species including eight LB species and ten RF species. Although it is uncontested that these differences exist between LB and RF *Borrelia*, we propose that the methodology used to identify these group-specific differences is subjective and has a highly limited power to delineate LB and RF *Borrelia* into separate genera.

The methodology employed by Adeolu and Gupta [14] reported only “CSIs that are specific for different groups within the *Borrelia*”, and CSPs only “if either all significant [BLAST] hits were from well-defined group of *Borrelia* or which involved a large increase in E-values from the last hit belonging to a particular group of *Borrelia* to the first hit from any other group”. This methodology specifically identifies only CSIs and CSPs that are exclusive only to one *Borrelia* genogroup, and precludes the detection of CSIs or CSPs that may be shared non-exclusively between both genogroups (i.e. contests the hypothesis that LB and RF belong in different genera). This data presented in isolation misrepresents the extent of genomic divergence between LB and RF *Borrelia* and fails to consider widespread genomic similarities between these two groups. Additionally, although ANI has previously been used to investigate prokaryote taxonomy [17], a comprehensive review of this method revealed that although ANI can accurately quantify the genetic relationships between strains belonging to the same species, it was not suitable to differentiate prokaryotic genera. This is due to significant overlapping of intergenera ANI and interspecies ANI values [18], which leads to unreliability in the method.

Alternatively, Qin et al. [18] presented a more heuristic method for delineating prokaryotic genera that measures the percentage of conserved proteins (POCP) between whole genome pairs, reasoning that the degree of protein conservation reflect both genetic and phenotypic relatedness more substantially. Qin et al. [18] demonstrated that among 235 prokaryotic species from 97 genera that POCP values had a higher predictive power than ANI to delineate genera. They showed that with few exceptions POCP values of ≥ 50% could be considered a threshold for prokaryotic genus delimitation, pending other genomic factors that influence POCP, such as large differences in genome size.

Here we investigate the validity of the proposed delineation of LB and RF *Borrelia* into separate genera by performing pairwise analysis of POCP values between 30 *Borrelia* type strain genomes (where possible), including two new *Borrelia* genomes from the novel reptile and echidna-associated clade, *B*. *turcica*, and *‘Candidatus* Borrelia tachyglossi’, which have yet to be analyzed in this context. We also re-examine the CSIs previously used to support the delineation of LB and RF *Borrelia* in the genomes of *B*. *turcica* and ‘*Candidatus* Borrelia tachyglossi’ to establish whether these molecular markers are useful to establishing the relationship of *B*. *turcica*, and *‘Candidatus* Borrelia tachyglossi’. Our analyses indicate that insufficient genomic divergence exist between LB and RF *Borrelia* to consider them separate genera, and that *Borrelia* CSIs are limited in their ability to unambiguously distinguish the taxonomic identity of *B*. *turcica* and ‘*Candidatus* Borrelia tachyglossi’.

## Materials and methods

### Strain included in this study

**Fig 1:**
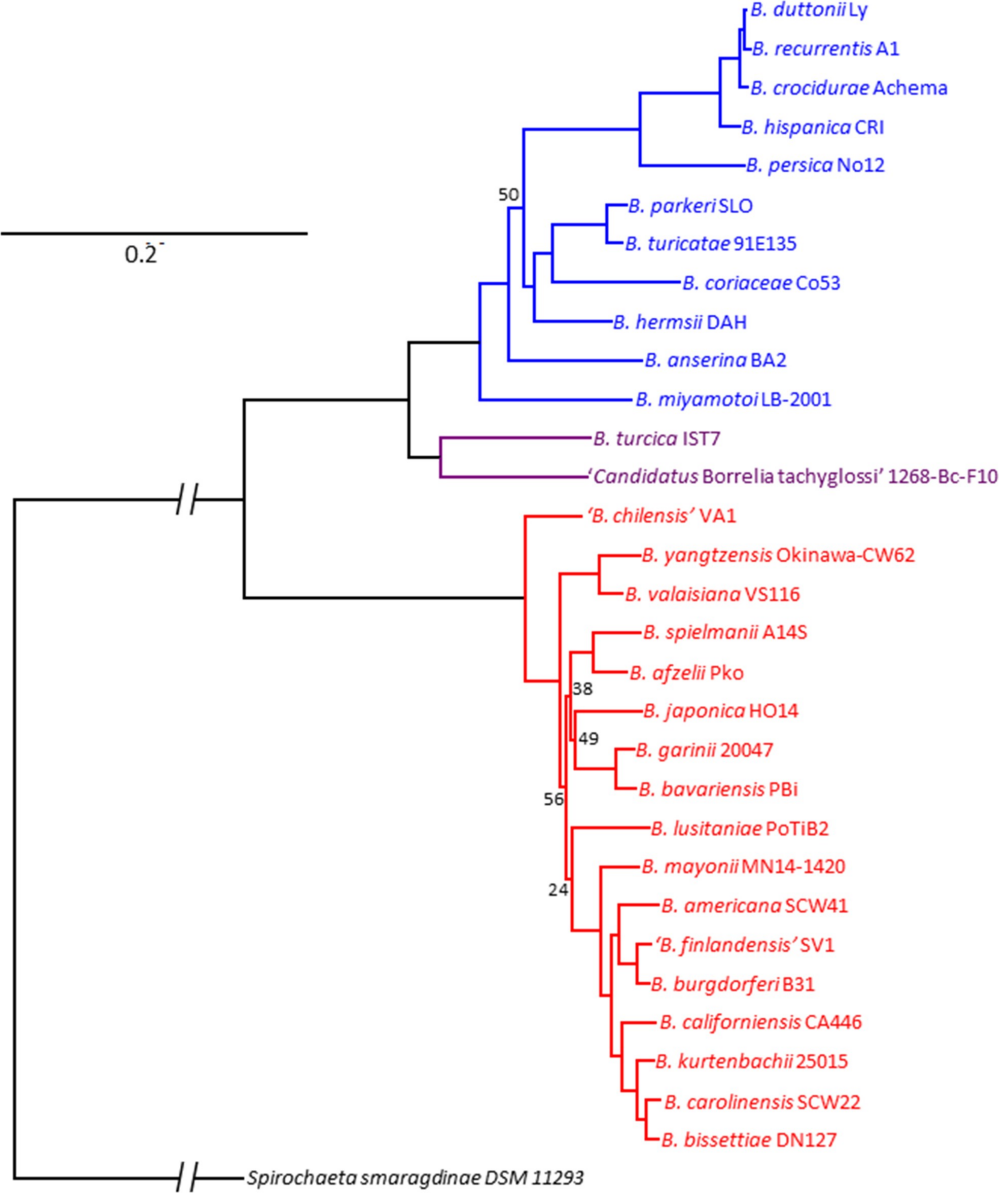
Phylogenetic reconstruction of *Borrelia* species based on 791 aligned protein homologs built with the PEPR pipeline and FastTree2 with 100 jackknifed resampling replicates. All node support values are 100 except where indicated.

In order to accurately assess *Borrelia* intra-genus POCP, the proteomes of 30 *Borrelia* species strains, including *n* = 17 strains from the LB group, *n* = 11 from the RF group, and *n* = 2 from the reptile and echidna-associated group, were retrieved from GenBank (National Center for Biotechnology Information (NCBI), Bethesda (MD), https://www.ncbi.nlm.nih.gov/) or sequenced and assembled from low passage type cultures except ‘*Candidatus* B. tachyglossi’ which was sequenced from a single tick [12, 19]. A tree summarizing the phylogenetic relationship based on 791 homologous proteins is shown in Fig 1. A full summary of strains used is presented in Table 1. To determine the levels of inter-genera POCP within the order Spirochaetales, an additional 54 proteomes, including *n* = 8 *Brachyspira*, *n* = 21 *Leptospira*, *n* = 5 *Spirochaeta*, and *n* = 20 *Treponema* species, were retrieved from GenBank (S1 Table) and included in the POCP analysis.

**Table 1:** Borrelia species and strains included in study.

	type strain	strain included	sequence source/GB accession number	available at culture collection			
* *				ATCC	DSMZ	CIP	JCM
**Lyme borreliosis group**							
*Borrelia afzelii* (Canica et al. 1994, sp. nov.)	VS461^T^	PKo	NC_008277		DSM-10508	CIP 103469	
*Borrelia americana* (Rudenko et al. 2010, sp. nov.)	SCW41^T^	SCW41 ^T^	SAMN05328445	BAA-1877	DSM-22541		
*Borrelia bavariensis* (Margos et al. 2013, sp. nov.)	PBi ^T^	PBi ^T^	CP028872	BAA-2496	DSM-23469		
*Borrelia bissettiae* (Margos et al. 2016, sp. nov.)	DN127 ^T^	DN127 ^T^	NC_015921		DSM-17990	CIP 109136	
*Borrelia burgdorferi* (Johnson et al. 1984, sp. nov.)	B31 ^T^	B31 ^T^	NC_001318	35210	DSM-4680		
*Borrelia californiensis* (Margos et al. 2016, sp. nov.)	CA446 ^T^	CA446 ^T^	SAMN05328472	BAA-2689	DSM-17989	CIP 109133	
*Borrelia carolinensis* (Rudenko et al. 2011, sp. nov.)	SCW22 ^T^	SCW22 ^T^	SAMN05328473	BAA-1773	DSM-22119		
*"Borrelia chilensis"*	VA1	VA1	CP009910				
*"Borrelia finlandensis"*	SV1	SV1	NZ_ABJZ00000000				
*Borrelia garinii* (Baranton et al. 1992, sp. nov.)	20047 ^T^	20047 ^T^	CP028861	51383	DSM-10534		
*Borrelia japonica* (Kawabata et al. 1994, sp. nov.)	HO14 ^T^	HO14 ^T^	SAMN05328497	51557			JCM 8951
*Borrelia kurtenbachii* (Margos et al. 2014, sp. nov.)	25015 ^T^	25015	SAMN05328498	BAA-2495	DSM-26572		
*Borrelia lusitaniae* (Le Fleche et al. 1997, sp. nov.)	PotiB2 ^T^	PotiB2 ^T^	SAMN05328499		DSM-107168	CIP 105366	
*Borrelia mayonii* (Pritt et al. 2016, sp. nov.)	MN14-1420 ^T^	MN14-1420 ^T^	NZ_CP015780	BAA-2743	DSM-102811		
*Borrelia spielmanii* (Richter et al. 2006, sp. nov.)	PC-Eq17 ^T^	A14S	NZ_ABKB00000000		DSM-16813	CIP 108855	
*Borrelia valaisiana* (Wang et al. 1997, sp. nov.)	VS116 ^T^	VS116 ^T^	SAMN02436326		DSM-21467	CIP 105367	
*Borrelia yangtzensis* (Margos et al. 2015, sp. nov.)	Okinawa-CW62 ^T^	Okinawa-CW62 ^T^	SAMN08904503	DSM-24625			JCM 17189
**Reptile associated group**							
*Borrelia turcica* (Güner et al. 2004, sp. nov.)	IST7 ^T^	IST7 ^T^	CP028884-91		DSM-16138		
**Australian *Borrelia***							
*Candidatus* Borrelia tachyglossi (Loh et al. 2017)		1268-Bc-F10*	CP025785-90				
**Relapsing fever group**							
*Borrelia anserina* (Sakharoff 1891) Bergey et al. 1925, species.	nd	BA2	NZ_CP005829				
*Borrelia coriaceae*	nd	Co53	NZ_CP005745	ATCC 43381			
*Borrelia crocidurae* (Leger 1917) Davis 1957, species.	nd	Achema	NC_017808				
*Borrelia duttonii* (Novy and Knapp 1906) Bergey et al. 1925, species.	nd	Ly	NC_011229				
*Borrelia hermsii* (Davis 1942) Steinhaus 1946, species.	nd	HS1	NZ_CP014349	BAA-2821	DSM 4682	CIP 104209	
*Borrelia hispanica* (de Buen 1926) Steinhaus 1946, species.	nd	CRI	NZ_AYOU00000000				
*Borrelia miyamotoi* Fukunaga et al. 1995	HT31^T^	LB-2001	NC_022079				
*Borrelia parkeri* (Davis 1942) Steinhaus 1946, species.	nd	SLO	NZ_CP005851				
*Borrelia persica* (Dschunkowsky 1913) Steinhaus 1946, species.	nd	No12	NZ_AYOT00000000				
*Borrelia recurrentis* (Lebert 1874) Bergey et al. 1925, species.	nd	A1	NC_011244				
*Borrelia turicatae* (Brumpt 1933) Steinhaus 1946, species.	nd	91E135	NC_008710				

GB = GenBank; ATCC = American Type Culture Collection; DSMZ = Deutsche Stammsammlung für Mikroorganismen und Zellkulturen; JCM = Japan Collection of Microoranisms; nd = no data

*sample ID

### Sequence analyses

POCP analysis was performed according to Qin et al. [18] and as described in [20]. Briefly, for each genome pair reciprocal BLASTP [21] was used to identify homologous proteins between genome pairs. Proteins were considered to be conserved if the BLAST matches had an E-value of < 1e-5, >40% sequence identity and >50% of the query sequence in each of the reciprocal searches. The POCP value for a genome pair was then determined as [(C_1_+C_2_)/(T_1_+T_2_)] x 100, where C_1_ and C_2_ are the number of conserved proteins between the genome pair, and T_1_ and T_2_ are the total number of proteins in each genome being compared [18]. Scripts used for these analyses are available upon request.

The CSIs presented in Adeolu and Gupta [14] that are differentially present in LB and RF genomes were reinvestigated in the genomes of *B*. *turcica* and ‘*Candidatus* Borrelia tachyglossi’ to establish whether these molecular markers are useful for classifying their taxonomic relationships. To identify CSIs, the conserved amino acid sequences flanking the CSIs were searched against the proteomes of all 30 *Borrelia* genomes used here using BLASTP [21]. Hits from the matching protein in all 30 *Borrelia* proteomes were aligned with MUSCLE [22], and visually inspected for the presence of CSIs. The presence of previously defined CSPs in the genomes of *B*. *turcica*, and *‘Candidatus* Borrelia tachyglossi’ was determined using BLASTP searched as described in Adeolu and Gupta [14].

## Results and discussion

In order to determine whether the 50% POCP threshold for genus delineation was appropriate for spirochete taxa, we used pairwise POCP analysis to determine the inter-genera POCP values for 84 spirochete genomes from the genera *Borrelia*, *Brachyspira*, *Leptospira*, *Spirochaeta*, and *Treponema*. Among all spirochete genomes investigated inter-genera POCP values ranged between 4.8% and 36.8% (mean 10.1%), indicating a low degree of protein conservation occurs between spirochete genera (S1 Table). These spirochete inter-genera POCP values are at a minimum of 13.2% lower than the 50% value determined by [18], suggesting this value is an appropriate and highly conservative threshold for spirochete genera delineation.

**Fig 2:**
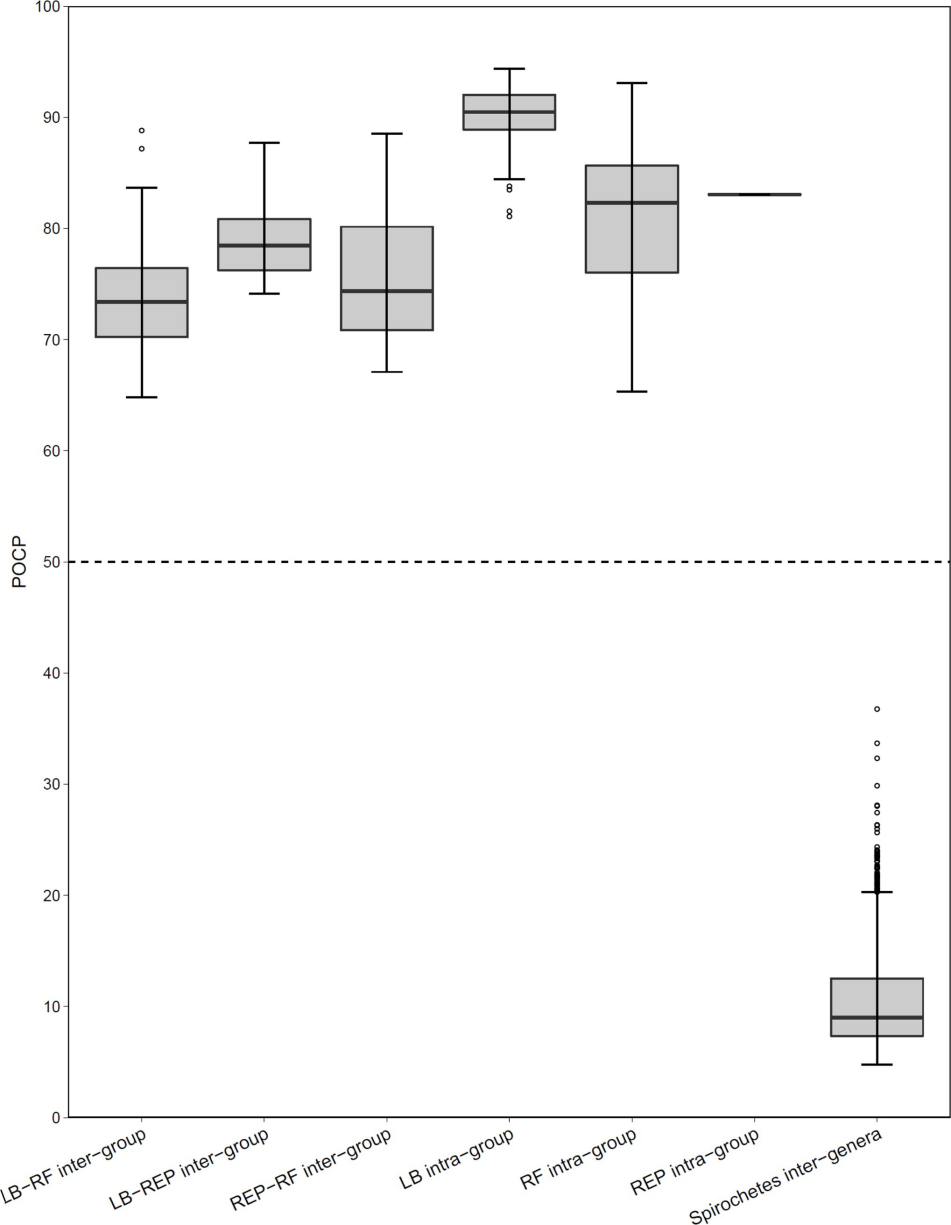
Boxplot of inter- and intra-specific POCP values. Inter-specific and intra-specific comparisons included Lyme borreliosis (LB) and relapsing-fever species (RF), reptile-associated species (REP) including the echnida-associated species ‘*Candidatus* B. tachyglossi’. The inter-genera comparison included the members of the genera *Borrelia*, *Brachyspira*, *Leptospira*, *Spirochaeta*, and *Treponema*.

**Fig 3:**
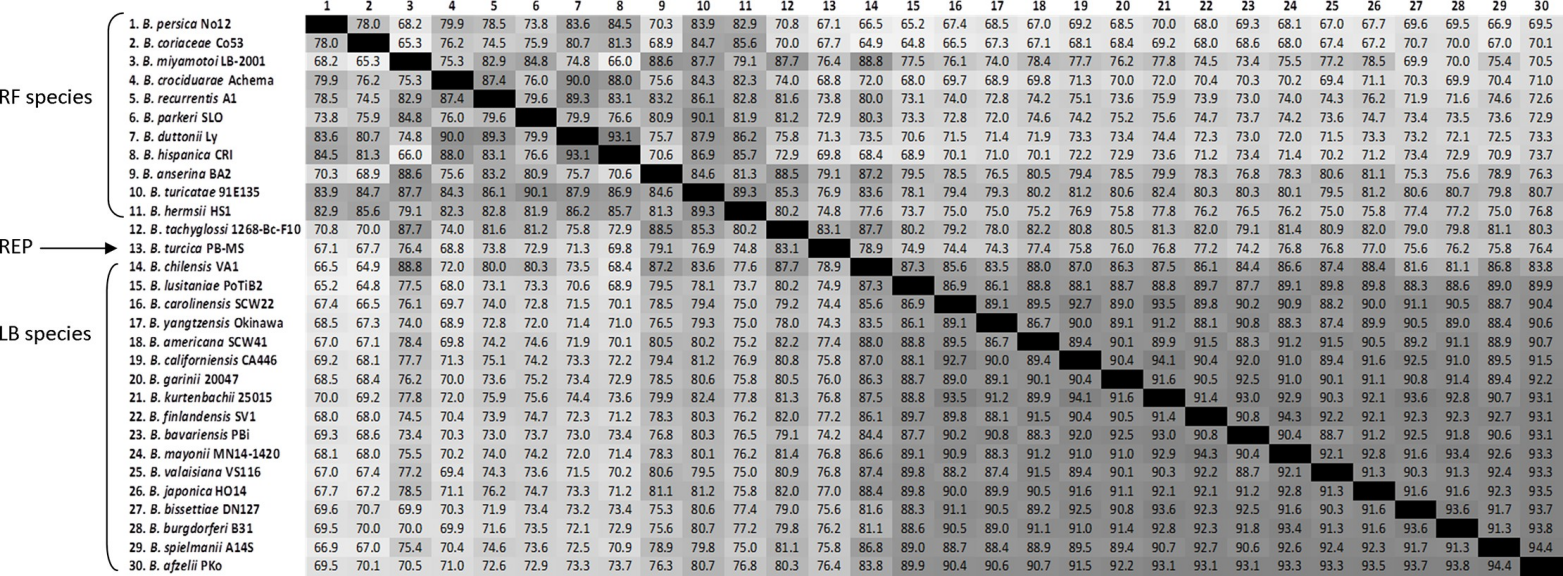
Percentage of conserved proteins (POCP) matrix generated by the method described in [18]. POCP values of species belonging to the LB group, RF group of spirochetes, the reptile-associated species *B*. *turcica* and echnida-associated species *B*. *tachyglossi* are above the genus threshold of 50%, indicating that all belong into one bacterial genus, *Borrelia*.

Compared to the low level of protein conservation measured between spirochete genera, POCP values were significantly higher among *Borrelia* species. *Borrelia* POCP values were highest among the LB genospecies, which ranged between 81.1–94.4% (mean 90.2%), while POCP values between RF species were generally lower and more variable, ranging between 65.3–93.1% (mean 81.1%) (Figs 2 and 3). Despite sharing a most recent common ancestor with RF *Borrelia*, *B*. *turcica* and ‘*Candidatus* Borrelia tachyglossi’ genomes shared higher POCP values with many LB *Borrelia* species compared to RF species, such as *B*. *chilensis* (78.9% and 87.7%, respectively), *B*. *americana* (77.4% and 82.2%, respectively), *B*. *japonica* (77.0% and 82.0%, respectively), and *B*. *mayonii* (76.8% and 81.42%, respectively). However, *B*. *turcica* and ‘*Candidatus* Borrelia tachyglossi’ genomes did have higher levels of protein conservation with *B*. *anserina* (79.1% and 88.5%, respectively) and *B*. *miyamotoi* (‘*Candidatus* Borrelia tachyglossi’ only: 87.7% POCP) (Figs 2 and 3). Most significantly, all *Borrelia* pairwise POCP values consistently remained well above the 50% POCP threshold for genus delineations proposed by Qin et al. [18], with a minimum value of 64.8% (*B*. *crocidurae* Achema vs. *B*. *chilensis* VA1), and a maximum value of 88.8% (*B*. *miyamotoi* LB-2001 vs. *B*. *chilensis* VA1) (mean value: 73.6%) (Figs 2 and 3).

**Fig 4:**
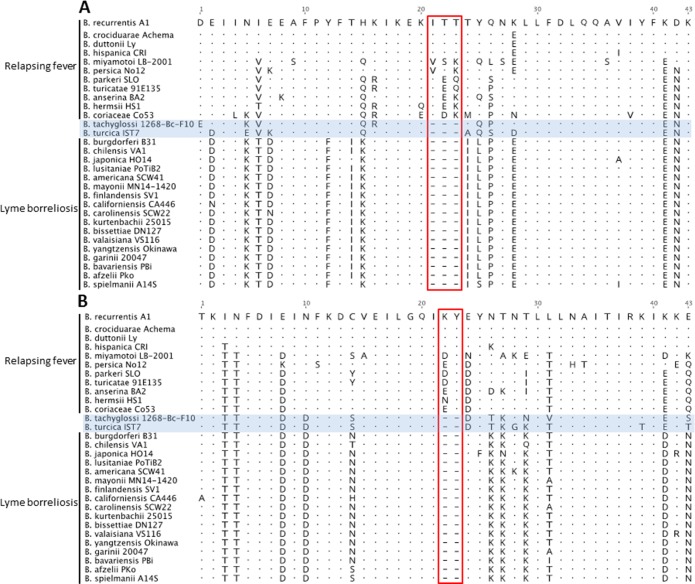
Partial amino acid alignment of (A) a putative lipoprotein (GI: 1195064) and (B) a hypothetical protein (GI: 1194969) showing a CSI in which the form of the indel in *‘Candidatus* Borrelia tachyglossi’ and *B*. *turcica* matches that in LB species.

The original proposal to delineate LB and RF *Borrelia* was largely based on the occurrence of 53 CSIs that have different forms in LB and RF genogroups [14]. It was subsequently defended by suggesting that novel *Borrelia* species that group with, or as an outgroup to RF *Borrelia* would be expected to contain RF-specific CSIs and generally none specific to the LB group [23]. An examination of these 53 CSIs in *B*. *turcica* and ‘*Candidatus* Borrelia tachyglossi’ genomes shows although the majority of CSIs present in these genomes correspond to the RF-specific form of the indels, 9/53 (17.0%) and 11/53 (20.8%) of the CSIs in *B*. *turcica* and ‘*Candidatus* Borrelia tachyglossi’, respectively, correspond to LB-specific forms (Fig 4; Table 2).

**Table 2:** Designation of LB and RF-differentiating CSIs in ‘*Candidatus* Borrelia tachyglossi’ and *B*. *turcica* genomes.

Gene	Size of CSI (aa)	‘*Candidatus* Borrelia tachyglossi’	*Borrelia turcica*
*RecA*	1	RF	RF
Nicotinamide-nucleotide adenylyltransferase	1	LB	LB
Hypothetical protein (BB0838)	3	LB	LB
Trigger factor *Tig*	2	RF	RF
Chemotaxis protein *CheY*	1	RF	RF
DNA polymerase III subunit beta	1	RF	RF
Translation factor Sua5	2	N/A	RF
Ferrous iron transporter	1	RF	RF
Glucose-6-phosphate isomerase	1	RF	RF
Hypothetical protein (BRE16)	3	RF	RF
Hypothetical protein (BDU327)	6	RF	RF
Hypothetical protein (BT0471)	1	LB	LB
L-latcate permease	1	RF	RF
1-phosphofructokinase	1	RF	RF
GTP-binding protein	2	RF	RF
Sodium/panthothenate symporter	1	LB	LB
Hypothetical protein (BRE32)	2	RF	RF
Hypothetical protein (Q7M33)	1	RF	RF
Hypothetical protein (BRE47)	5	RF	RF
L-proline transport system ATP-binding protein	1	RF	RF
Penicillin-binding protein	1	RF	RF
Hypothetical protein (Q7M131)	1	RF	RF
Hypothetical protein (BT0110)	2	RF	RF
Hypothetical protein (BB0110)	2	RF	RF
Glutamate racemase	6	RF	RF
16S riboisonal RNA methyltransferase *RsmE*	1	RF	RF
DNA mismatch repair protein *mutL*	3	RF	RF
Putative lipoprotein	3	LB	LB
Membrane protein	1	LB	RF
Hypothetical protein (BRE314)	1	RF	RF
Methylgalactoside ABC transporter ATP-binding protein	1	RF	RF
Hypothetical protein (BRE355)	1	LB	LB
Sensor transduction histidine kinase	1	RF	RF
DNA polymerase III subunit delta	2	LB	RF
Hypothetical protein (Q7M860)	2	RF	RF
Hypothetical protein (KK90081)	1	RF	RF
Hypothetical protein (Q7M140)	2	LB	LB
Hypothetical protein (BG0159)	1	LB	LB
Outer membrane protein	1	RF	RF
Transglycosylase SLT domain-containing protein	1	RF	RF
Cell division protein *FtsZ*	1	RF	RF
Excinuclease ABC subunit C	1	RF	RF
Hypothetical protein (BG0519)	1	RF	RF
Hypothetical protein (BBIDN1270545)	5	RF	RF
Hypothetical protein (BBUN400354)	3	RF	RF
Hypothetical protein (BBUZS70553)	1	RF	RF
Hypothetical protein (BB0554)	1	RF	RF
Hypothetical protein (BB0554)	2	RF	RF
Hypothetical protein (BBUCA803285)	1	RF	RF
Methyl-accepting chemotaxis protein	2	LB	LB
Chemotaxis protein	1	RF	RF
Chemotaxis protein	1	RF	RF
Hypothetical protein (L14403475)	1	RF	RF

Thus, the results of our analysis using genospecies that were originally defined as belonging to the genus *Borrelia* showed a very clear pattern. The results demonstrate that LB and RF *Borrelia* genogroups lack sufficient proteomic differentiation to be classified as different genera according to the POCP threshold determined by Qin et al. [18]; the analysis of inter-genus POCP supported the classification of the five closely related Spirochaetales genera. Therefore, we propose to formally reestablish the genus *Borrelia* in its original form including species of the LB, RF, and reptile- and echnida-associated genogroups. Additionally, up to 20% of the CSIs identified as having genogroup-specific forms were not concordant with phylogenetic position of *B*. *turcica* and ‘*Candidatus* Borrelia tachyglossi’ as predicted previously [23]. Although categorical molecular markers such as these have been previously used in clarifying prokaryotic taxonomy, here these markers appear to have limited utility in resolving the taxonomic classification of novel *Borrelia* species.

The reptile- and echidna-associated *Borrelia* clade to which *B*. *turcica* and ‘*Candidatus* B. tachyglossi’ belong is a very recently described group of *Borrelia* for which several novel variants have been described based on single- or multi-gene phylogenetic analyses. Although this group clearly shared a more common ancestor with RF *Borrelia*, the presence of LB-specific CSIs and high protein conservation with LB species suggests this *Borrelia* may share common genetic and biological characteristics with LB species. Both, PCOP and CSI supported the continuum of *Borrelia* species between LB and RF which now includes *B*. *turcica* and ‘*Candidatus* B. tachyglossi’. These data suggest that the genus *Borrelia* in the form it was originally described and is proposed here represents a continuum with RF and LB group species at the extreme ends of the genus, and reptile and echnida-associated, and other *Borrelia* species (perhaps still to be discovered) sharing a unique mixture of features from both RF and LB groups.

In our study we included as many type strains as possible, as type strains are the representatives of the species and can be obtained from microbial culture collections. However, for two of the species belonging to the LB group of spirochetes, genomic data of the type strains were not available to us. As a surrogate we used genomic data available for closely related strains of these species, i.e. PKo for *B*. *afzelii* and A14S for *B*. *spielmanii*. Previous data on multilocus sequence typing have shown that these two isolates are closely related to the type strain of the respective genospecies and fall into the same phylogenetic cluster [24, 25].

We consider that *Borreliella bavariensis* (Margos *et al*. 2013) Adeolu and Gupta 2015, *Borreliella burgdorferi* (Johnson et al. 1984) Adeolu and Gupta 2015, *Borreliella carolinensis* (Rudenko et. al 2011) Adeolu and Gupta 2015, *Borreliella garinii* (Baranton et al. 1992) Adeolu and Gupta 2015, *Borreliella japonica* (Kawabata et al. 1994) Adeolu and Gupta 2015, *Borreliella kurtenbachii* (Margos et al. 2014) Adeolu and Gupta 2015, *Borreliella sinica* (Masuzawa et al. 2001) Adeolu and Gupta 2015, *Borreliella spielmanii* (Richter et al. 2006) Adeolu and Gupta 2015 should be more appropriately placed in the genus *Borrelia* as *Borrelia bavariensis* Margos *et al*. 2013, *Borrelia burgdorferi* Johnson et al. 1984, *Borrelia carolinensis* Rudenko et. al 2011, *Borrelia garinii* Baranton et al. 1992, *Borrelia japonica* Kawabata et al. 1994, *Borrelia kurtenbachii* Margos et al. 2014, *Borrelia sinica* Masuzawa et al. 2001, *Borrelia spielmanii* Richter et al. 2006 which we consider to be the correct name of the taxon. *Borreliella bavariensis* (Margos *et al*. 2013) Adeolu and Gupta 2015, *Borreliella carolinensis* (Rudenko et. al 2011) Adeolu and Gupta 2015, *Borreliella garinii* (Baranton et al. 1992) Adeolu and Gupta 2015, *Borreliella japonica* (Kawabata et al. 1994) Adeolu and Gupta 2015, *Borreliella kurtenbachii* (Margos et al. 2014) Adeolu and Gupta 2015, *Borreliella sinica* (Masuzawa et al. 2001) Adeolu and Gupta 2015, *Borreliella spielmanii* (Richter et al. 2006) Adeolu and Gupta 2015 should be considered to be synonyms.

## Conclusion

The data presented in this study very clearly demonstrate that all groups investigated, i.e. RF group spirochetes, LB group spirochetes, reptile- and echnida-associated *Borrelia* species belong to the same genus as values for POCP were consistently above the proposed threshold for genus delimitation. We propose to re-establish the genus *Borrelia* in its original form.

**Emended description of the genus Borrelia** (Swellengrebel 1907) (approved lists 1980)

Organisms are helical (0.2–0.3 μm by 10–35 μm). Periplasmic flagella overlap in the central region of the cell. Cells are flexible and motile with rotational and forward/backwards movement. Organisms are host-associated and microaerophilic. They are vectored by argasid ticks, prostriate ixodid ticks, metastriate ixodid ticks and the human body louse. The genome is fragmented into a linear main chromosome, and linear or circular plasmids. The G/C content of the genomic DNA is 27–32 (mol%).

Members of this genus are the causative agents of relapsing fever, Lyme borreliosis or of unknown pathogenicity.

The type species is *Borrelia anserina* (Sakharoff 1891) Bergey *et al*. 1925(Approved Lists 1980).

**Description of *Borrelia afzelii*** Canica et al 1994

The description is the same as in Canica et al 1994. DNA-DNA hybridization, RFLP of the *rrs* gene as well as reactivity of monoclonal antibodies differentiates *B*. *afzelii* from other Borrelia species [26, 27]. *B*. *afzelii* strains are also distinguishable from all other LB species by using Multilocus sequence analysis [24].

Type strain VS461^T^ (= DSM 10508^T^ = CIP 103469^T^)

**Description of *Borrelia lusitaniae*** (Le Fleche et al. 1997, sp. nov.)

The description is the same as given in Le Fleche et al. 1997.

Type strain PotiB2 ^T^ (= CIP-105366 ^T^, = DSM-107168^T^)

## Supporting information

S1 Table Additional proteomes from the order Spirochaetales included in this study. A distance matrix of POCP values is given for genera included and shows that the within genus percentages are generally higher than 50% except for *Treponema*.
(XLSX)
